# P227: Outbreak of Serratia marcescens in a postoperative cardiosurgery unit in a tertiary care hospital

**DOI:** 10.1186/2047-2994-2-S1-P227

**Published:** 2013-06-20

**Authors:** M Grande, I Wijers, P Navarro, V Nováková, C Bové, P Rodríguez

**Affiliations:** 1 Preventive Medicine and Quality Management, General University Hospital Gregorio Marañón. Madrid. Spain

## Introduction

We report an outbreak of infections with *S. Marcescens* in patients on a Postoperative Cardiosurgery Unit (PCU), and describe the epidemiological investigations and control measures undertaken.

## Methods

After the identification of various microbiological results positive for *S*. m*acescens* in cultures from patients admitted to the PCU, active surveillance through clinical and microbiological results was initiated. Retrospectively, we searched for *S*. m*arcescens* positive microbiological results from patients that had been admitted to the PCU in the preceding months. All patients staying on the PCU were screened for *S*. *marcescens* colonization. Environmental samples were taken from the PCU as well as from the operating room where the patients had been operated. *S. marcescens* strains were identified.

## Results

In the month of August, after moving the PCU and the cardiac surgery operating room to temporary locations due to renovation works, three patients, admitted to the PCU, were identified to have cultures positive for *S. marcescen*s. One of them died from a septic shock due to bacteremia secondary to central line infection with *S*. *marcescens*. The other two patients presented bacteremic ventilator-associated pneumonia. Specific precautions based on contact transmission were implemented and the staff was instructed about hand hygiene with alcohol-based hand rub. Retrospectively, two more patients with *S*. *marcescens* positive cultures were identified. One of them, who had had a diagnosis of bacteremic mediastinitis, had been discharged from the original PCU location. The second patient, who had been admitted in the same period, had a wound culture positive for *S. marcescen*s. This second patient had been moved to the new location and was still staying in the PCU. Neither the environmental samples (soaps, antiseptics, saline solutions, catheters…) from the PCU, nor the samples taken from the operating room were positive for *S. marcescens*. It was confirmed that in all of the cases it was the same *S. marcescens* strain.

## Conclusion

Data suggested that the mode of transmission was most likely due to the transfer of organisms from person to person by cross transmission.

## Disclosure of interest

None declared

